# Predictors of maternal residential mobility in a sibling-matched birth cohort in Massachusetts

**DOI:** 10.1097/EE9.0000000000000452

**Published:** 2026-01-07

**Authors:** Jesselle M. Legaspi, Veronica M. Vieira

**Affiliations:** a Department of Environmental and Occupational Health, Joe C. Wen School of Population & Public Health, University of California, Irvine, California

**Keywords:** Residential mobility, Environmental exposure, PM_2.5_, Maternal health, Pregnancy, Birth cohort, Socioeconomic status

## Abstract

**Background:**

Residential mobility during pregnancy and between births is common and can introduce exposure misclassification. It may also reflect broader sociodemographic and environmental inequalities that influence maternal and child health. The objective of this study is to evaluate associations between maternal sociodemographic characteristics, PM_2.5_ exposure, and residential mobility. Among mothers who moved, additional analyses evaluated predictors of relocation to lower-income census tracts.

**Methods:**

Data were obtained from 155,270 mothers with matched sibling birth records from the Massachusetts Pregnancy to Early Life Longitudinal data system (2001–2009). We define residential mobility as a change in geocoded birth addresses and identified relocation to a lower-income census tract among movers. PM_2.5_ exposure estimates at each birth address were assigned using annual averages from a previously validated spatiotemporal model. Logistic regression was used to examine associations between residential mobility and maternal age, race/ethnicity, education, parity, and residential PM_2.5_ exposure.

**Results:**

Among mothers with linked births, 49.3% moved between births, and 19.9% relocated to a census tract with a lower median income. Mothers that moved between births had significantly lower PM_2.5_ at the subsequent birth address compared with mothers that did not move. Compared with mothers living at low PM_2.5_ exposure levels (5th percentile, 1 µg/m^3^), mothers living at high PM_2.5_ exposure levels (95th percentile, 11 µg/m^3^) had a nearly three-fold higher odds of moving. Relocation to a lower-income tract was less likely among older mothers, non-Hispanic mothers, and those with more than a high school education.

**Conclusion:**

Environmental and sociodemographic factors shape residential mobility patterns between births. It is important to account for residential mobility to reduce exposure misclassification and improve accuracy in perinatal epidemiology.

**What this study adds**

Many pregnant women move between births, but these moves are not random. This study shows how factors like age, race, education, and air pollution levels shape where mothers live over time. By tracking address changes in a large sibling birth cohort in Massachusetts, we find who is most likely to move to lower-income neighborhoods and how PM_2.5_ differs for those who move. Our findings highlight the importance of accounting for residential history to reduce exposure misclassification in environmental epidemiology.

## Introduction

Maternal residential mobility, defined as the movement of mothers and their families from one residence to another, can have significant implications for family stability, health, and environmental and socioeconomic exposures.^1,2^ Mothers often experience multiple moves during pivotal life stages, such as pregnancy, between the births of children, and while raising children, which can disrupt both environmental and social stability.^3–5^ Frequent relocations during these stages can complicate assessments of long-term environmental and socioeconomic exposures, potentially affecting both maternal and child health.^2–4^ Understanding the factors that drive maternal residential mobility is crucial, as it is shaped by a range of social, economic, and environmental influences, including maternal age, race, education, and socioeconomic status.^3,6,7^

Residential mobility during the time between the births of children can introduce variability in socioeconomic and environmental exposures, leading to changes in household income, neighborhood resources, or exposure to environmental risks.^2,4^ Families may move due to growing family size, job changes, or economic instability, which can either improve or worsen the conditions mothers and their children experience.^2,8,9^ For many low-income families, residential mobility is driven by eviction, unstable housing, or financial distress, often resulting in repeated moves within disadvantaged areas that reinforce socioeconomic instability.^8^ Low-income women are particularly vulnerable to frequent moves during pregnancy due to financial hardship or limited housing options, often relocating to neighborhoods with poorer healthcare and education, less green space, and higher pollution levels.^8,10–12^ In contrast, higher-income families have greater control over their housing choices, experience fewer moves, and benefit from greater residential stability.^13^

Educational attainment significantly influences residential mobility, with higher education generally protective against moves to lower-income neighborhoods.^3,7,11^ Mothers with higher educational attainment tend to experience housing stability due to greater economic resources and access to stable housing environments.^11,14^ Conversely, lower educational attainment is associated with more frequent moves, often due to limited financial resources and fewer opportunities for stable, long-term housing.^9^ Research shows that frequent residential mobility can disrupt family routines and affect children’s educational outcomes, especially in lower-income households, where moves are often accompanied by school changes.^5,9,15^ These disruptions can negatively impact children’s academic performance, particularly in urban settings where mobility rates are higher, and access to quality education is more limited.^9^ As such, educational attainment not only influences the stability of maternal housing but also the broader socioeconomic environment in which children are raised.

Younger maternal age is associated with a higher likelihood of moving during pregnancy, a pattern often driven by economic instability and life changes that tend to occur earlier in adulthood.^3,16^ In addition to age, race and ethnicity significantly influence mobility patterns, particularly for Black and Hispanic families, who face higher rates of moves to lower-income neighborhoods due to persistent structural inequalities and housing discrimination.^17,18^ These barriers often restrict access to stable, high-quality housing and contribute to ongoing cycles of residential instability and economic hardship.^17^ Such patterns of mobility have been linked to adverse outcomes for maternal and child health, including increased stress, reduced access to healthcare, and disruptions in childhood development.^1,19^

Maternal residential mobility during pregnancy can impact health outcomes and complicate the assessment of environmental exposures.^4^ Epidemiological studies often rely on the maternal residence at delivery as a proxy for exposure throughout pregnancy, but this can lead to misclassification, as an estimated 20%–30% of pregnant women move at least once.^3,4^ This is particularly relevant for air pollutants such as fine particulate matter (PM_2.5_), which can vary significantly across locations.^4,20^ PM_2.5_, particulate matter with a diameter of 2.5 micrometers or smaller, is a significant environmental health concern.^21–23^ Particles in the PM_2.5_ size range and finer have the ability to penetrate deep into the respiratory system, causing a variety of health problems.^24,25^ Pregnant women and their developing fetuses, and growing children, are especially vulnerable to these pollutants, with exposure linked to adverse birth outcomes such as low birth weight, preterm birth, and neurodevelopmental diseases.^26–28^ These associations with adverse health outcomes can motivate decisions to relocate, often resulting in moves to areas with different socioeconomic profiles.^29,30^ Understanding the predictors of mobility between births may offer insights into which populations are more likely to move and under what circumstances, helping to identify when and where exposure misclassification is most likely to occur. By identifying the factors driving mobility, such as age, socioeconomic status, and family size, researchers can refine exposure assessments, ensuring more accurate measurements across different stages of pregnancy and child-rearing in future studies.

This study aims to investigate the associations between sociodemographic and environmental factors on residential mobility patterns using data from the Pregnancy to Early Life Longitudinal (PELL) data linkage system in Massachusetts from 2001 to 2009. By linking births from the same mother over time, this approach examines how sociodemographic factors (i.e., age, education, income, and number of siblings) and environmental exposures such as PM_2.5_ can influence residential mobility patterns for mothers and their children. Recognizing the factors that influence mobility is essential for minimizing bias in exposure assessments in epidemiology. Moreover, accurate assessments are critical for informing policies and interventions aimed at promoting maternity and family stability, particularly in vulnerable populations where frequent moves may intensify existing socioeconomic challenges.

## Methods

### Study population

This study involves secondary analysis of deidentified data from the PELL data linkage system; thus, Institutional Review Board approval was not necessary. The PELL system is a comprehensive data resource combining birth and fetal death files with hospital discharge records of both mothers’ delivery and infants’ births in Massachusetts. Additionally, it is a partnership between the Boston University School of Public Health, the Massachusetts Department of Public Health, and the Centers for Disease Control and Prevention.^31^ Analysis is conducted under a data use agreement in accordance with all applicable ethical guidelines and data protection regulations. Mothers were assigned a unique ID, allowing all of their Massachusetts births to be longitudinally linked. These data have been used previously to identify sibling-matched controls.^32^

The PELL dataset included 693,018 total births for 2001–2009. We excluded 1.5% (n = 10,346) due to missing geocoded birth addresses. We then removed duplicate birth records for multiple births (e.g., twins or triplets), resulting in 666,652 unique pregnancies. Multiple births were only included if additional birth records from separate pregnancies were recorded. We then restricted the sample to mothers with more than one pregnancy in the PELL dataset for 2001–2009, resulting in 339,201 birth records. For mothers with more than two separate births, only the first and second birth records were selected for a total of 310,540 birth records linked to 155,270 mothers for analysis.

### Data collection

Individual-level maternal sociodemographic characteristics, including age, race/ethnicity, and education attainment, were obtained from birth records in the PELL dataset. Geocoded maternal residential addresses for each birth were linked to census tract-level median household income data from the American Community Survey.^6,32^

The PM_2.5_ measurements used in this study were provided by Girguis et al.^32^ Briefly, the PM_2.5_ exposure assessment involved a multistage modeling approach that estimated PM_2.5_ using a spatiotemporal model that combined satellite-derived aerosol optical depth data, meteorological data, and land-use variables. Daily PM_2.5_ concentrations were estimated at a 1 × 1 km spatial resolution.^33–36^ Maternal addresses were geocoded and linked to the corresponding annual average PM_2.5_ exposure estimates based on birth year.

### Outcome assessment

Residential mobility was defined as a move from the maternal address reported on the initial birth record to a different address for the subsequent birth record in the PELL data linkage. Among those who moved, a secondary outcome assessed whether the move was to a census tract with a lower median income.

### Predictors

The choice of predictors was informed by previous research on residential mobility. Maternal age at the subsequent PELL birth was included as a continuous variable. We used age at subsequent birth rather than age at initial birth as we believe it is likely closer to the age the mother moved. Additional continuous variables included the median household income of the census tract and PM_2.5_ concentrations at the initial and subsequent birth addresses. Race/ethnicity (non-Hispanic white, non-Hispanic black, Hispanic, Asian/Pacific Islander, and other), education level (less than high school, high school graduate, and more than high school), and parity of the mother (less than 2 children, 2 or more children) at the subsequent birth were modeled categorically. These factors were chosen based on their established associations with residential mobility in similar studies.^1,6,37^

### Statistical analysis

Our main objective was to investigate the predictors of maternal residential mobility. We tested the differences between movers and nonmovers using two-sample *t*-tests for continuous variables and chi-square tests for categorical variables. The odds of a mother moving between PELL-linked births were modeled using logistic regression models and controlled for maternal age, race/ethnicity, education, and parity.

To assess which were better predictors of moving, the attributes of the home they were moving from or the attributes of the home they were moving to, we fit two separate models with census-tract median income and PM_2.5_ concentrations, one with measures at the initial address and the second with measures for the subsequent address. The model for the initial address provided information about what factors may influence moving away from that residence, whereas the model for the subsequent address identified factors that may influence moving to a new location.

We report odds ratios (OR) and 95% confidence intervals (CI) of moving between births and living in addresses with a difference of $10,000 in a census-tract median household income. We also predict ORs of moving between births for different combinations of income, education, and age (at 5th, 50th, and 95th percentiles) to assess the intersectionality of these predictors (e.g., for a mother living in a high-income census tract (95th percentile) with less than a high school degree compared with a mother living in a median-income census tract with some college education). For PM_2.5_ concentrations, we report the OR (95% CI) per 10 µg/m³ increase, which represents the risk of moving for a mother who lives in a low exposure area (5th percentile) compared with a mother who lives in a high exposure area (95th percentile).

In a secondary analysis among mothers that moved, we were interested in predictors of moving to a lower-income census tract. Similarly, the effects of PM_2.5_ concentrations at the different addresses were modeled in two separate models, and all models adjusted for maternal age, race/ethnicity, education, and parity. Descriptive statistics were calculated for all covariates. Mothers with missing individual-level data for maternal race/ethnicity, education, or parity (n = 1,007) were excluded from regression models. In addition, we excluded mothers for which census tract-level median household income could not be assigned (n = 62). Statistical analyses were conducted in R version 4.0.3 (R Foundation for Statistical Computing, Vienna, Austria).

## Results

Of the 155,270 mothers with linked PELL birth records and geocoded addresses from 2001 to 2009, 76,487 (49.3%) moved between births, and 30,902 (19.9%) moved to a census tract with a lower median income. Table 1 presents the demographic and socioeconomic characteristics stratified by mobility status. Mothers primarily identified as non-Hispanic white (65.6% of movers and 79.1% of nonmovers). Mothers who moved had a lower mean age of 29.6 years than those who did not move, with a mean age of 32.0 years. In terms of education, mothers who did not move were more likely to have completed more than a high school education (76.4%) compared with those who moved (58.2%). In contrast, movers had a higher proportion of mothers with lower educational attainment where 12.4% had not completed high school and 29.5% are high school graduates, compared with 5.2% and 18.3%, respectively, among nonmovers. As shown in Table 1, all reported characteristics differed significantly between movers and nonmovers (*P* < 0.001).

**Table 1.** Maternal demographic and socioeconomic characteristics of mothers with linked PELL births (2001–2009) in Massachusetts, stratified by residential mobility

		Nonmovers		Movers	
Characteristic^a^		N	Percentage (%)	N	Percentage (%)
Total		78,783		76,487	
Maternal age (years)^b^	Mean ± SD	32.0 ± 5.1		29.6 ± 5.7	
	Median (5th, 95th percentile)	33 (22; 40)		30 (20; 39)	
Maternal race/ethnicity	Non-Hispanic White	62,332	79.1	50,134	65.6
	Non-Hispanic Black	4,209	5.3	7,007	9.2
	Hispanic	6,279	8.0	12,767	16.7
	Asian/Pacific Islander	4,898	6.2	4,842	6.3
	Other	986	1.3	1,654	2.2
	Missing	79	0.1	83	0.1
Maternal education	Less than high school	4,056	5.2	9,208	12.0
	High school graduate	14,429	18.3	22,589	29.5
	More than high school	60,175	76.4	44,534	58.2
	Missing	123	0.2	156	0.2
Parity	<2 siblings	59,167	75.1	54,977	71.9
	2 or more siblings	19,304	24.5	21,256	27.8
	Missing	312	0.4	254	0.3
Median income of the census tract of the initial PELL birth address (USD)	Mean ± SD	76,120 ± 34,622		63,789 ± 32,078	
	Median (5th; 95th percentile)	73,081 (26,193; 138,162)		59,753 (18,963; 121,563)	
Median income of the census tract of the subsequent PELL birth address (USD)	Mean ± SD	76,916 ± 34,659		68,576 ± 36,290	
	Median (5th; 95th percentile)	73,805 (26,875; 139,464)		63,913 (19,099; 135,132)	
PM_2.5_ exposure of initial PELL birth address (µg/m³)	Mean ± SD	10.4 ± 1.7		10.8 ± 1.6	
	Median (5th; 95th percentile)	10.5 (7.9; 12.7)		10.8 (8.4; 12.9)	
PM_2.5_ exposure of subsequent PELL birth address (µg/m³)	Mean ± SD	8.1 ± 3.2		7.7 ± 3.5	
	Median (5th; 95th percentile)	9.1 (1.0; 11.1)		9.0 (1.0; 11.1)	
Time between births (years)	Mean ± SD	2.4 ± 1.2		3.1 ± 1.6	
	Median (5th; 95th percentile)	2.0 (1.0; 5.0)		3.0 (1.0; 6.0)	

a All differences between movers and nonmovers are statistically significant (*P* < 0.001), based on two-sample *t*-tests for continuous variables and chi-square tests for categorical variables.

b Maternal age, race/ethnicity, education, and parity are reported from the address at the subsequent PELL birth record.

In addition, movers resided in census tracts with a lower median income at their initial address ($63,789) compared with nonmovers ($76,120). Movers also experienced slightly higher PM_2.5_ exposure at their initial address (10.8 µg/m³) compared with nonmovers (10.4 µg/m³). After moving, movers had a lower PM_2.5_ exposure (7.7 µg/m³) than nonmovers (8.1 µg/m³) in the year and address of their subsequent PELL birth. Movers had a longer time between births (3.1 years) compared with nonmovers (2.4 years). Among all movers, the correlation between initial and subsequent census tract median income was r = 0.66, while the correlation between initial and subsequent PM_2.5_ exposure was r = 0.47, indicating moderate continuity in neighborhood socioeconomic and environmental conditions due to spatial and temporal differences across moves. In comparison, temporal changes alone among nonmovers resulted in more correlated measures with r = 0.81 for income and r = 0.46 for PM_2.5_ between births.

Table 2 describes characteristics of movers by income direction of the move. Mothers who moved to lower-income census tracts had a younger mean age (28.6 years) compared with those who moved to non-lower-income census tracts (30.3 years). Additionally, 60.3% of mothers who moved to lower-income census tracts identified as non-Hispanic white, compared with 69.1% for those who moved to higher-income tracts.

**Table 2.** Maternal demographic and socioeconomic characteristics of mothers who moved between initial and subsequent PELL births (2001–2009) in Massachusetts, stratified by whether the move was to a lower-income census tract

		Movers to a census tract of equal or higher income		Movers to a lower-income census tract	
Characteristic^a^		N	Percentage (%)	N	Percentage (%)
Total		45,585		30,902	
Maternal age (years)^b^	Mean ± SD	30.3 ± 5.6		28.6 ± 5.7	
	Median (5th; 95th percentile)	31 (21; 39)		28 (20; 38)	
Maternal race/ethnicity	Non-Hispanic White	31,505	69.1	18,629	60.3
	Non-Hispanic Black	3,558	7.8	3,449	11.2
	Hispanic	6,467	14.2	6,300	20.4
	Asian/Pacific Islander	3,130	6.9	1,712	5.5
	Other	882	1.9	772	2.5
	Missing	43	0.1	40	0.1
Maternal education	Less than high school	4,522	9.9	4,686	15.2
	High school graduate	11,809	25.9	10,780	34.9
	More than high school	29,166	64.0	15,368	49.7
	Missing	88	0.2	68	0.2
Parity	<2 siblings	33,020	72.4	21,957	71.1
	2 or more siblings	12,411	27.2	8,845	28.6
	Missing	154	0.3	100	0.3
Median income of the census tract of the initial PELL birth address (USD)	Mean ± SD	56,050 ± 28,837		75,198 ± 33,191	
	Median (5th; 95th percentile)	52,054 (16,500; 107,232)		71,212 (30,521; 135,568)	
Median income of the census tract of the subsequent PELL birth address (USD)	Mean ± SD	81,564 ± 36,770		49,413 ± 25,477	
	Median (5th; 95th percentile)	77,115 (31,103; 148,947)		45,625 (14,726; 96,583)	
PM_2.5_ exposure of initial PELL birth address (µg/m³)	Mean ± SD	10.8 ± 1.6		10.7 ± 1.6	
	Median (5th; 95th percentile)	10.9 (8.4; 12.9)		10.8 (8.3; 12.8)	
PM_2.5_ exposure of subsequent PELL birth address (µg/m³)	Mean ± SD	7.9 ± 3.2		7.4 ± 3.9	
	Median (5th; 95th percentile)	8.9 (1.0; 11.0)		9.0 (1.0; 11.1)	
Time between births (years)	Mean ± SD	3.1 ± 1.5		3.2 ± 1.6	
	Median (5th; 95th percentile)	3..0 (1.0; 6.0)		3.0 (1.0; 6.0)	

a All differences between movers to a census tract of equal or higher income and those to a lower-income census tract are statistically significant (*P* < 0.001), based on two-sample *t*-tests for continuous variables and chi-square tests for categorical variables.

b Maternal age, race/ethnicity, education, and parity are reported from the address at the subsequent PELL birth record.

Education levels also varied, with 49.7% of those moving to lower-income census tracts having more than a high school education, compared with 64.0% for those moving to higher-income areas. As expected, the median income of the census tract at the subsequent birth address is significantly lower for mothers who moved to lower-income census tracts ($49,413) compared with those who moved to higher-income tracts ($81,564).

In terms of air pollution exposure, the PM_2.5_ levels at the initial address for movers to lower-income census tracts were 10.7 µg/m³, similar to levels (10.8 µg/m³) for movers to higher-income tracts. However, at the subsequent address, the PM_2.5_ exposure is lower for movers to lower-income census tracts (7.4 µg/m³) than for those moving to higher-income census tracts (7.9 µg/m³). The difference is not likely due to temporal trends in air pollution. The average time between births is similar for mothers who moved to lower-income census tracts compared with those who moved to higher-income census tracts (3.2 vs. 3.1 years). Of movers to lower-income census tracts, the correlation between initial and subsequent census tract income was r = 0.74, and the correlation in PM_2.5_ exposure was r = 0.65, indicating relatively high continuity in both income and pollution levels despite downward mobility. For movers to equal or higher-income tracts, the income correlation was slightly lower (r = 0.73), with a weaker correlation in PM_2.5_ exposure (r = 0.47).

Table 3 presents the OR for moving versus not moving between initial and subsequent PELL birth records. A 10 µg/m³ increase in PM_2.5_ exposure is associated with a 195% higher likelihood of moving away from the initial birth address (adjusted OR = 2.95, 95% CI = 2.75, 3.15). In contrast, a $10,000 increase in the median income of the census tract at the initial address is associated with a 5% decreased likelihood of moving away (adjusted OR = 0.95, 95% CI = 0.95, 0.96), indicating more residential stability in higher-income neighborhoods.

**Table 3.** Odds ratios and 95% confidence intervals for the association between maternal and residential characteristics and moving between PELL births (2001–2009) in Massachusetts

Characteristics^a^	Adjusted OR (95% CI)
Maternal age^b^	0.74 (0.72, 0.75)
Maternal race/ethnicity	
Non-Hispanic White	Reference
Non-Hispanic Black	1.69 (1.62, 1.76)
Hispanic	1.59 (1.53, 1.65)
Asian/Pacific Islander	1.20 (1.15, 1.25)
Other	1.63 (1.51, 1.78)
Maternal education	
Less than high school	Reference
High school graduate	0.90 (0.86, 0.94)
More than high school	0.60 (0.58, 0.63)
Parity	
<2 siblings	Reference
2 or more siblings	1.18 (1.15, 1.21)
Median income of the census tract of the initial PELL birth address^c^	0.95 (0.95, 0.96)
Median income of the census tract of the subsequent PELL birth address^c^	1.02 (1.01, 1.02)
PM_2.5_ exposure (µg/m³) of initial PELL birth address^d^	2.95 (2.75, 3.15)
PM_2.5_ exposure (µg/m³) of subsequent PELL birth address^d^	0.70 (0.68, 0.73)

a Maternal age, race/ethnicity, education, and parity are reported using the address at the subsequent PELL birth record.

b Per 5-year increase in maternal age.

c The effect estimates for the median income of the census tract are calculated for a $10,000 increase.

d The effect estimates for PM_2.5_ exposure are calculated for a 10 µg/m³ increase.

Contrastingly, for predictors measured at the subsequent birth address, a 10 µg/m³ increase in PM_2.5_ exposure is associated with a 30% lower likelihood of moving to that location (adjusted OR = 0.70, 95% CI = 0.68, 0.73), suggesting selective avoidance of more polluted locations. A $10,000 increase in the median income of the census tract at the subsequent address is only associated with a 2% increase in the odds of moving to that higher-income area (adjusted OR = 1.02, 95% CI = 1.01, 1.02).

Maternal age at the subsequent birth is inversely related to the odds of moving, with a 5-year increase in age being associated with a 26% decrease in the odds of moving (adjusted OR = 0.74, 95% CI = 0.72, 0.75). Racial and ethnic differences are evident in mobility patterns. Compared with non-Hispanic white mothers, non-Hispanic black mothers had 69% higher odds of moving (adjusted OR = 1.69, 95% CI = 1.62, 1.76), Hispanic mothers had 59% higher odds of moving (adjusted OR = 1.59, 95% CI = 1.53, 1.65), and Asian/Pacific Islander mothers had 20% higher odds of moving (adjusted OR = 1.20, 95% CI = 1.15, 1.25). Mothers identifying as “Other” races had 63% higher odds of moving compared with non-Hispanic white mothers (adjusted OR = 1.63, 95% CI = 1.51, 1.78).

Education level is a significant predictor of residential mobility. Mothers with more than a high school education had 40% lower odds of moving compared with those with less than a high school education (adjusted OR = 0.60, 95% CI = 0.58, 0.63). Those with a high school education had a 10% lower likelihood of moving compared with those with less than a high school education (adjusted OR = 0.90, 95% CI = 0.86, 0.94). Parity, or the number of children a mother had, also influenced moving to a new residence. Mothers with two or more prior births had an 18% higher likelihood of moving compared with those with less than 2 prior births (adjusted OR = 1.18, 95% CI = 1.15, 1.21).

Table 4 presents the predicted odds of residential mobility through the intersectionality of maternal education with percentiles for age and the median income of their census tract, while holding other covariates at their median. Mothers with less than a high school education had the highest predicted odds of mobility at younger ages (OR = 2.08, 95% CI = 2.06, 2.10 for age 21 years) and lower household income (OR = 1.03, 95% CI = 1.02, 1.04 at $21,552) compared with a mother that is 31 years old with a high school education and median income, while those with more than high school education had lower odds across all strata (lowest OR = 0.41, 95% CI = 0.41, 0.41 for age 39).

**Table 4.** Adjusted odds ratios for moving between PELL births predicted for different combinations of education level, maternal age and census tract income

Education	Age		Income		Odds	Adjusted OR (95% CI)
Value	Percentile	Value	Percentile	Value		
<HS	5th%	21	50th%	69,519	2.18	2.08 (2.06, 2.10)
<HS	95th%	39	50th%	69,519	0.69	0.68 (0.67, 0.69)
>HS	5th%	21	50th%	69,519	1.29	1.25 (1.25, 1.26)
>HS	95th%	39	50th%	69,519	0.42	0.41 (0.41, 0.41)
<HS	50th%	31	5th%	21,552	1.06	1.03 (1.02, 1.04)
<HS	50th%	31	95th%	136,667	1.28	1.24 (1.23, 1.26)
>HS	50th%	31	5th%	21,552	0.64	0.62 (0.62, 0.63)
>HS	50th%	31	95th%	136,667	0.77	0.75 (0.74, 0.75)
HS	50th%	31	50th%	69,519	1.03	–

The OR for moving to a lower-income census tract versus an equal or higher-income census tract among movers is presented in Table 5. Among mothers who moved, higher PM_2.5_ exposure at both the initial and subsequent PELL birth addresses was significantly associated with lower odds of relocating to a lower-income census tract. A 10 µg/m³ increase in PM_2.5_ exposure at the initial birth address was associated with a 39% decreased odds of moving away from the equal or higher-income tract (adjusted OR = 0.61, 95% CI = 0.55, 0.67), whereas higher PM_2.5_ levels at the subsequent address were associated with a 33% lower odds of moving into a location of a lower-income census tract (adjusted OR = 0.67, 95% CI = 0.64, 0.70).

**Table 5.** Odds ratios and 95% confidence intervals for the association between maternal and residential characteristics and moving to a lower-income census tract among mothers who moved between PELL births (2001–2009) in Massachusetts

Characteristics^a^	Adjusted OR (95% CI)
Maternal age^b^	0.83 (0.82, 0.84)
Maternal race/ethnicity	
Non-Hispanic White	Reference
Non-Hispanic Black	1.44 (1.37, 1.51)
Hispanic	1.23 (1.18, 1.28)
Asian/Pacific Islander	0.94 (0.88, 1.00)
Other	1.25 (1.13, 1.39)
Maternal education	
Less than high school	Reference
High school graduate	0.99 (0.94, 1.04)
More than high school	0.72 (0.68, 0.76)
Parity	
<2 siblings	Reference
2 or more siblings	1.08 (1.04, 1.17)
PM_2.5_ exposure (µg/m³) of initial PELL birth address^c^	0.61 (0.55, 0.67)
PM_2.5_ exposure (µg/m³) of subsequent PELL birth address^c^	0.67 (0.64, 0.70)

a Maternal age, race/ethnicity, education, and parity are reported using the address at the subsequent PELL birth record.

b Per 5-year increase in maternal age.

c The effect estimates for PM_2.5_ exposure are calculated for a 10 µg/m³ increase.

Compared with non-Hispanic white mothers, non-Hispanic Black mothers had 44% increased odds of moving into a lower-income census tract (adjusted OR = 1.44, 95% CI = 1.37, 1.51), while Hispanic mothers had 23% increased odds (adjusted OR = 1.23, 95% CI = 1.18, 1.28). Each 5-year increase in maternal age at the subsequent birth record was associated with a 17% decrease in the odds of moving to a lower-income tract (adjusted OR = 0.83, 95% CI = 0.82, 0.84).

Educational attainment showed mixed associations with residential mobility to lower-income census tracts. Having a high school diploma as the highest level of education is not significantly associated with the odds of moving to a lower-income area (adjusted OR = 0.99, 95% CI = 0.94, 1.04). In contrast, mothers with more than a high school education had 38% decreased odds of relocating to a lower-income census tract compared with those with less than a high school education (adjusted OR = 0.72, 95% CI = 0.68, 0.76). Conversely, higher parity is associated with slightly increased odds of moving to a lower-income area, with mothers who had two or more prior births exhibiting an 8% higher likelihood compared with those with less than 2 prior births (adjusted OR = 1.08, 95% CI = 1.04, 1.17).

## Discussion

This analysis identifies key sociodemographic and environmental predictors of maternal residential mobility between births and highlights the potential for misclassification of exposure if the birth address is used rather than the address during pregnancy. Younger maternal age, lower educational attainment, and higher parity are associated with a greater likelihood of moving, particularly to lower-income census tracts. Movers had lower PM_2.5_ exposures compared with nonmovers in the year and address of their subsequent PELL birth, suggesting that using birth addresses for exposure assessment may result in underestimation of exposure if mothers moved during pregnancy. Interestingly, movers to lower-income neighborhoods observed lower PM_2.5_ exposures at the new address compared with PM_2.5_ exposures of those who moved to equal or higher income census tracts, suggesting a complex relationship between socioeconomic and environmental factors in residential decision-making.

Maternal age at the subsequent birth emerged as a strong and consistent predictor of residential mobility. Younger mothers were more likely to relocate between births than older mothers, consistent with prior studies showing that younger maternal age is linked to higher residential instability due to economic constraints, unstable relationships, or transitions in employment and housing.^3,12,38,39^ This age-related pattern may introduce bias into environmental exposure studies if younger mothers disproportionately relocate in response to environmental stressors or socioeconomic instability.

Maternal educational attainment and parity play a similarly crucial role in predicting residential mobility patterns. Mothers with limited education were more likely to move between births, while mothers with higher education had a lower likelihood of moving into lower-income neighborhoods. This aligns with literature showing that educational attainment correlates with greater housing security and access to desirable neighborhoods.^3,9,14^ Higher-educated mothers may have the financial and social resources to avoid such downward moves, consistent with evidence that education enables families to reside in safer, less polluted environments.^18^ While it is possible that adjusting for maternal education and census-tract median household-income in the same model may over-adjust for the effect of socioeconomic status, neither variable alone is accurate enough to fully capture the effect of income, which could result in results being impacted by residual confounding.

Predicted OR from our model further demonstrates this intersectionality of risk factors. Among mothers with less than a high school education, the risk of moving was three times higher for mothers of younger age (5th percentile) compared with older mothers (95th percentile). Additionally, for mothers living in census tracts with higher median household income, the odds of moving were higher if they had lower education compared with some college. Our results further underscore the importance of considering age and income together with education.

In terms of parity, some studies report greater residential mobility among younger, primiparous women.^16^ However, results of this analysis found that mothers with multiple children had a slight increase in the likelihood of moving to lower-income tracts than first-time mothers. This was likely driven by economic pressures of larger households that prompt moves to more affordable and potentially higher-pollution areas.^40^

Disparities by race and ethnicity are also evident and consistent with established literature on residential stratification. Non-Hispanic Black and Hispanic mothers are significantly more likely to move to lower-income neighborhoods compared with non-Hispanic white mothers. Structural forces such as historical redlining, residential segregation, and housing discrimination constrain neighborhood choices for racially marginalized populations.^17,41,42^ These inequities extend beyond income and race/ethnicity but often intersect with environmental burdens. However, we observe higher PM_2.5_ exposure at the initial birth address to be associated with decreased odds of moving to a lower-income neighborhood. While lower-income and racially segregated neighborhoods typically face higher levels of ambient air pollution due to proximity to highways, industrial sources, and fewer regulator protections.^37,43–45^ The observed inverse association of air pollution exposure may be explained by urban areas or certain neighborhoods with high traffic-related pollution, while also being relatively affluent, such as parts of Boston’s South End or Back Bay. It may also be possible that higher-pollution neighborhoods, such as urban centers, offer affordable housing, access to transportation, or social services that discourage relocation.

These findings suggest that disparities in air pollution exposure are shaped not only by income and race but also by structural constraints that limit the ability to relocate, especially for communities of color. Even after accounting for socioeconomic status, racially marginalized groups were more likely to remain in higher-pollution environments,^46^ which could reflect the constrained choice to move. Therefore, recognizing structural constraints and their interactions with socioeconomic status, race and ethnicity, and environmental conditions can accurately capture the contexts of exposures and avoid misclassification, particularly when relocation patterns are driven by factors beyond an individual’s choice.

In addition to environmental exposures, neighborhood-level income also influenced residential mobility. An increase in the median income of the census tract at the initial PELL birth address is associated with lower odds of moving away from that location, consistent with prior findings that higher-income neighborhoods offer greater residential stability due to better resources.^1,47,48^ In contrast, an increase in the median income at the subsequent address is associated with higher odds of having moved to that higher-income area, supporting theories of upward residential mobility and neighborhood selection.^49–51^ These findings suggest that neighborhood income promotes stability while simultaneously attracting new residents.

The observed associations between PM_2.5_ exposure and residential mobility suggest that air pollution may motivate relocation. Higher PM_2.5_ levels at the initial address were associated with a nearly three-fold higher odds of moving away. Conversely, increased PM_2.5_ at the subsequent address was associated with a 30% lower likelihood of moving to that location, suggesting selective avoidance of more polluted neighborhoods. However, it is also possible that only mothers with greater resources can avoid high-exposure neighborhoods, while less advantaged mothers may be constrained to remain in them.^52,53^

These findings have important implications for exposure assessment in environmental and perinatal epidemiology. Many maternal and infant health studies assign exposure based on maternal residence at the time of delivery, assuming housing stability throughout pregnancy and infancy. However, approximately 20%–30% of pregnant individuals move at least once^3,4^ and the current analysis confirms that residential mobility patterns were shaped by age, education, race, income, and environmental context. Therefore, using the delivery address alone may lead to systematic exposure misclassification. For instance, mothers exposed to higher PM_2.5_ may move before delivery, leading to underestimates of exposure if only the delivery address is accounted for. If more educated or economically advantaged mothers are more likely to move to less polluted areas, while disadvantaged mothers remain in high-exposure locations, the resulting exposure estimates could be differentially biased by maternal characteristics.

This form of differential misclassification could either attenuate or exaggerate true exposure-outcome associations, depending on the direction and nature of the bias. Several studies have demonstrated that failing to account for residential mobility during pregnancy introduces nonrandom error in exposure assessment, particularly when mobility is associated with sociodemographic factors.^4,7,54^ For example, Hodgson et al^4^ found that residential moves during pregnancy led to exposure misclassification for air pollutants like PM_2.5_, with the degree of error depending on the pollutant’s spatial resolution. Canfield et al^54^ reported that 31%–33% of women changed residence between conception and delivery, with younger age and lower income associated with higher residential mobility, suggesting sociodemographic patterns in misclassification. Ling et al^7^ further showed that residential mobility during early childhood significantly impacts estimates of exposure to pesticides, with reduced accuracy in models using only a single address.

Such misclassification is especially problematic in studies of exposures with high spatial heterogeneity (e.g., traffic-related air pollution or pesticides), where distance moved and location-specific variability can critically alter exposure history.^16,55^ Pennington et al^55^ demonstrated that even small residential movements altered modeled air pollution exposure enough to bias risk estimates. Our findings reinforce the importance of incorporating residential histories or applying correction methods when assessing prenatal environmental exposures. Without such adjustments, exposure assessment remains vulnerable to bias, particularly in perinatal epidemiology, where timing and location of exposure are critical to outcome risk.

Although this study did not examine birth outcomes, the observed residential mobility patterns have implications for maternal and child health. Moving to or from high-exposure neighborhoods across pregnancies can result in unmeasured changes in environmental risk, which if unmeasured, can distort associations in longitudinal or sibling-based studies. Prior research shows that air pollution is associated with adverse birth outcomes such as low birth weight, preterm birth, and neurodevelopmental effects.^21,55,56^ This issue is especially critical in studies of spatially and temporally variable exposures such as PM_2.5_, where such misclassification could attenuate real associations or introduce spurious ones.^34,36^ Therefore, capturing accurate residential histories across gestation is essential for assessing cumulative exposure and environmental risks more precisely. When full address history is not available, imputation strategies or probabilistic models could be used to estimate likely patterns of residential movement and adjust exposure assignments accordingly.^57–59^

In conclusion, this analysis highlights the social and environmental factors driving maternal residential mobility, such as age, education, race/ethnicity, neighborhood income, and air pollution levels. Importantly, we demonstrated that mobility patterns are not random and vary across subgroups, which has direct implications for how exposure is assigned and interpreted in perinatal research. Studies that assign exposure based solely on the maternal address at delivery and ignore residential mobility can result in differential exposure misclassification, leading to under- or overestimation of exposures within specific subpopulations and obscuring true health disparities. Future research should incorporate residential histories across gestation to better characterize time-weighted exposures. Understanding who moves, where, and why is critical for refining exposure assessment methodologies and reducing bias in environmental health risks estimates, particularly in populations where housing patterns intersect with disparities in environmental exposures.

## Conflicts of interest statement

The authors declare that they have no conflicts of interest with regard to the content of this report.

## Acknowledgments

The authors thank the Massachusetts Department of Public Health and Boston University School of Public Health for access to the PELL data system. The contents of this manuscript are solely the responsibility of the authors and do not necessarily represent the official views of the Massachusetts Department of Public Health or Boston University.
